# Isokinetic Knee Muscle Strength Parameters and Anthropometric Indices in Athletes with and without Hyperextended Knees

**DOI:** 10.3390/medicina60030367

**Published:** 2024-02-22

**Authors:** Sasa Bascevan, Barbara Gilic, Mirela Sunda, Marijana Geets Kesic, Petra Zaletel

**Affiliations:** 1 Clinic Patella, 10000 Zagreb, Croatia; sasa.bascevan@kif.unizg.hr; 2 Faculty of Kinesiology, University of Split, 21000 Split, Croatia; markes@kifst.hr; 3 High Performance Sport Center, Croatian Olympic Committee, 10000 Zagreb, Croatia; 4 Faculty of Kinesiology, University of Osijek, 31000 Osijek, Croatia; msunda@kifos.hr; 5 Faculty of Sport, University of Ljubljana, 1000 Ljubljana, Slovenia; petra.zaletel@fsp.uni-lj.si

**Keywords:** hypermobile joints, joint health, physical therapy, sports performance, sports rehabilitation

## Abstract

*Background and Objectives*: Hypermobility has been linked to decreased knee performance, including isokinetic and isometric knee strength. This study aimed to determine whether athletes with and without knee hyperextension have different hamstring-to-quadriceps strength (H/Q) ratios and to investigate the associations between knee hyperextension indices and H/Q ratios and anthropometric characteristics. *Materials and Methods*: The sample consisted of 47 healthy male athletes without knee injuries aged 23.48 ± 3.54 years. The variables included the degree of knee hypermobility, isokinetic parameters of the leg musculature, and anthropometric indices. Differences between athletes with and without hyperextension were calculated using an independent sample *t*-test, effect sizes, and discriminant analysis, while associations between the variables were checked by Pearson’s correlation coefficient and multiple regression analysis. *Results*: Athletes with hyperextended knees had shorter legs (t value = −2.23, *p* = 0.03, moderate ES) and shins (t = −2.64, *p* = 0.01, moderate ES) and a lower H/Q ratio at an angular velocity of 60°/s (t = −2.11, *p* = 0.04, moderate ES) than those in the nonhyperextended group did; these differences were supported by discriminant analysis (Wilks’ L = 0.60, *p* = 0.01). An increase in the H/Q ratio at an angular velocity of 60°/s was associated with the degree of knee hypermobility (R = −0.29, *p* = 0.04). *Conclusions*: This research showed that athletes with knee hypermobility have weaker hamstring strength and thus a lower H/Q strength ratio at lower angular velocities. These findings suggest that targeted strength training programs for leg (i.e., hamstrings) muscles should help individuals with knee hypermobility.

## 1. Introduction

Hypermobility, or excessive joint laxity, is a common and clinically significant finding in the treatment of musculoskeletal diseases [1]. Generalized joint hypermobility (GJH) refers to the capacity of the joint to stretch beyond the usual physiological range of motion (ROM) and is simply described as synovial joint hyperextensibility [1,2]. The prevalence of GJH in adults ranges from 5% to 43%, while the prevalence in children ranges from 2% to 55%, which is determined by the method used for classifying GJH [3]. GJH can be present alone, in conjunction with symptoms (e.g., pain, exhaustion, or joint dislocations), or as a characteristic of a well-defined condition, such as inherited connective tissue disorders. The commonly used method for determining hypermobility analyzes five parts of the body where hypermobility is easily recognized, namely, the little finger, thumb, elbow, knee, and spine [4]. Indeed, hypermobility has been linked to ankle sprains, anterior cruciate ligament injuries, shoulder instability, and hand osteoarthritis. Patients with hypermobility and musculoskeletal injuries frequently seek treatment for generalized musculoskeletal pain and injuries that are not clearly caused by the disorder [5].

Hypermobility is widespread in young patients and is connected with an increased risk of musculoskeletal injury [5]. It is assumed that GJH reduces joint stability, increasing the likelihood of joint and soft tissue injuries during athletic activity [6]. Indeed, individuals with GJH are more likely to sustain dislocations, subluxations, and sprains during physically demanding activities, especially those involving the lower limbs [2,3,7]. Moreover, a review study that evaluated the influence of GJH and the risk of lower limb injuries during sports noted that joint hypermobility has been most commonly linked to a greater likelihood of lower-limb injuries, particularly knee ligament injuries [3]. Namely, it is generally accepted that hypermobility and particularly hyperextension of the knee joint are the most significant risk factors for noncontact injuries, such as the most common knee injury, anterior cruciate ligament (ACL) injury [1,2].

Hyperextension of the knee or hypermobile knee (lat. genu recurvatum—saber leg), is characterized by overextension of the knee of 10° or more than full extension. When standing still, it is characterized by a hypotonized quadriceps muscle and a stretched biceps femoris muscle [8]. Furthermore, the hypermobile knee is characterized by somewhat more stretched cruciate ligaments and greater pressure from the femoral condyles on the menisci and tibial condyles when standing, which may explain the phenomenon in which people with hypermobile knees are unable to stand for a long time without feeling pain or discomfort [9]. Additionally, patients with GJH and hyperextended knees reported a lower health-related quality of life [10]. The theory that a higher total body mass and body mass index will be positively correlated with hyperextension of the knee in such a way that in the support phase there will be more pressure on the back structures of the knee and thus overextend the knee is not proved. Indeed, a study of 96 girls aged 16 to 18 showed that there is no correlation between body mass index and knee hypermobility [11], while a study of 420 children aged 6 to 12 indicated that hypermobility is more common in children who have below average body weight (57%), while only 17% of hypermobile children had above average body weight [12]. Also, it is theorized that longer legs will undergo greater force, resulting in higher mobility degree, but such theory is not proven.

It is believed that the development of muscles, especially the ratio of agonistic to antagonistic muscle groups around the joint, is important for joint health [7,13]. Moreover, an imbalance in strength and hypotrophy of the knee joint flexor and extensor muscles are muscular malfunctioning determinants that change joint stability and predispose athletes to injury [14]. The hamstrings-to-quadriceps strength (H/Q) ratio is considered an important indicator of imbalances in muscle strength around the knee joint and is used for detecting injury risk [15]. Specifically, the H/Q ratio is usually determined by an isokinetic device, which measures muscle fore at a specified constant joint angular velocity (e.g., 60°/s, 180°/s). According to numerous investigations performed over the last three decades, the absolute quadriceps muscle force (knee extension) should exceed the hamstring muscle force (knee flexion) by a magnitude of 3:2, which represents an H/Q ratio of 0.66 [16]. In other words, the consensus for the normative value of the H/Q ratio for injury prevention by detecting muscle imbalances is at most 0.6 [17]. Indeed, a low H/Q ratio was associated with several severe knee injuries, including hamstring strains and ACL injuries [18,19]. Given that isokinetic tests at higher angular speeds better describe muscle activity at speeds at which the knee is used during sports activities, the authors consider higher angular speeds and the results achieved in them as functional indicators, while those achieved at lower angular speeds are considered standard indicators. Therefore, this study included measurements mostly linked to standard knee activity (angular velocity of 60°/s and 180°/s) [20].

Hypermobility has been linked to decreased knee performance, including decreased proprioception and isokinetic and isometric knee strength [21]. However, there is limited evidence on muscle activation in hypermobile individuals, making it unclear whether functional impairments and the requirement for joint stabilization in GJH are linked to muscle activity [13]. Hypermobility is more common in females than in males and is generally accepted as one of the leading risk factors for knee ligament injuries [10]. Therefore, the majority of studies have focused on investigating hypermobility and its determinants in females, while fewer investigations have been conducted exclusively on males and without the presence of knee injuries [10,22,23]. Moreover, there is a lack of research regarding the association between the H/Q strength ratio and hypermobility indices in healthy male athletes. Therefore, the primary aim of this study was to determine whether athletes with and without knee hyperextension have different H/Q strength ratios. The secondary aim of this study was to investigate the associations between knee hyperextension indices and H/Q strength ratios at different joint angular velocities and between knee hyperextension indices and anthropometric characteristics in athletes. The hypothesis of this study is that athletes with knee hyperextension will have less favorable H/Q strength ratios and that hyperextension indices will be negatively correlated with H/Q strength ratios.

## 2. Materials and Methods

### 2.1. Participants

The sample consisted of 47 healthy male athletes without knee injuries aged 23.48 ± 3.54 years, with a height of 182.02 ± 7.03 cm and a body mass of 80.27 ± 10.5 kg. Athletes from various sports were included, from soccer (44.68%), fitness training (10.64%), track and field (8.51%), rowing (8.51%), basketball (8.51%), combat sports (6.39%), handball (4.25%), swimming (4.25%), tennis (2.13%), and volleyball (2.13%). Athletes were divided into two groups: athletes with knee hypermobility (10° or more than full knee extension) and athletes without knee hypermobility. The exclusion criterion was any history of knee injury within the last 6 months which included muscle, ligament, and tendon injuries. Additionally, all potential participants who, in the last three months, performed certain forms of isolated exercise for the muscle groups included in the test, especially those for the hamstrings, were excluded from the test. This was set as the exclusion criterion because the significant effect of such exercises was connected to the results of isokinetic testing in terms of increasing the maximum values of the hamstrings, which affects the ratio of the tested muscles [24]. Also, included athletes must not have previously been tested on isokinetic devices of any kind (due to the effect of repeated testing on results). From the group of hypermobile athletes, no selection was made for sports activity, so that the influence of sports activity on the results would not be hidden (so there may have been some swimmers or gymnasts, but they did not dominate). To clarify, participants were collected randomly; the call for participating in the testing was spread across social media, through orthopedic clinics, sports clubs, and other sports organizations. Thus, the study included all participants who were actively involved in sports, who suspected they had hypermobility, and all those who were interested in such testing regardless of the mobility status. Naturally, more persons without hypermobility volunteered to join, but the researchers limited their number when they reached the targeted number of individuals. The sample presents participants from various sports as the authors did not want to exclusively include athletes more prone to hypermobility issues (e.g., swimmers, gymnasts). Collectively, the study’s sample represents a convenient sample of healthy athletes from various sports, without any lower-limb injuries or deformities.

### 2.2. Variables and Testing Procedures

The variables in this research included (in the testing order) anthropometric indices, the degree of knee hypermobility, and isokinetic parameters of leg musculature (hamstrings and quadriceps muscles) in an open kinetic chain.

The anthropometric indices included body height (expressed in centimeters), body mass (expressed in kilograms), body mass index (BMI), and leg dimensions. The leg length was measured with an anthropometer such that the subject stood with his legs extended, while the measurer placed the lower arm at the point of support with the base. The anthropometer was placed perpendicular to the base, and the upper arm was placed on the lateral proximal tip of the upper leg. To measure the length of the shin, the subject stood upright while the researcher measured the length of the lower leg with a shortened anthropometer so that the lower leg was placed exactly on top of the lateral malleolus and the upper leg was placed exactly on the top of the lateral condyle of the tibia. The measurements are expressed in centimeters.

Knee hypermobility was measured (degree of knee hypermobility—Hdeg) by the subject sitting on the floor, while legs were stretched with heels placed on a bench 20 cm high. The tester placed the goniometer (Pasco PS-2002 Xplorer GLX, Roseville, CA, USA) in the center of rotation of the knee and directed one extended arm toward the lateral proximal tip of the upper leg and the other toward the lateral malleolus of the same leg. The same procedure was used for the other leg.

Isokinetic testing was conducted using a Biodex System 4 isokinetic dynamometer (Biodex Medical Systems, Inc., Shirley, NY, USA) by an experienced researcher. The individuals were fastened with belts around the upper body and upper leg of the hips, the axis of the dynamometer was directed to the area above the lateral condyle of the femur, and the bandage on the lower leg was placed just above the lateral malleolus. The axis of the dynamometer was placed at the point of rotation of the knee, which could be additionally checked by extending and bending the subject’s knee. Before the measurement, the subject received detailed instructions about the test and the specifics of the isokinetic movement. It should be emphasized that during the actual testing, the participant was vocally motivated to perform to the maximum extent throughout the entire testing. The tests included concentric–concentric tests at an angular velocity of 60°/s and concentric–concentric tests at an angular velocity of 180°/s. All isokinetic testing was conducted by the same experienced tester/researcher to reduce the measurement error.

For the purposes of this research, the subjects were measured in a range of motions of 70 degrees so that, in the initial position, the lower leg was placed vertically against the floor. The reason for this lies in the fact that an amplitude of 90 degrees, when the body is placed upright in a sitting position, can cause the subject to be unable to perform full movement due to the possibly insufficiently flexible muscles in the back of the upper leg, which causes them to perform an isometric contraction instead of the final movement [25]. Similarly, given that the research required only the maximum result in extension and flexion (peak torque), i.e., their ratio, and given that the maximum result in isokinetic measurements at a speed of 60 degrees per second occurs at 54 degrees from the start of extension (quadriceps) and 33 degrees from the start of knee flexion (hamstrings), it was not necessary to test with full amplitude. At an angular velocity of 180 degrees per second, the maximum result occurs on average at approximately 43 degrees of extension and 40 degrees of flexion [25].

**Figure 1:**
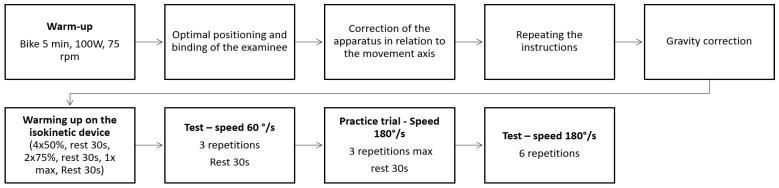
Testing protocol for the isokinetic variables.

The isokinetic variables included in this research were the H/Q ratio of the right leg at 60°/s (H/Q60R), the H/Q ratio of the left leg at 60°/s (H/Q60L), the average H/Q ratio at 60°/s for both legs (H/Q60), the H/Q ratio of the right leg at 180°/s (H/Q180R), and the H/Q ratio of the left leg at 180°/s.—(H/Q180L), and the average H/Q ratio was 180°/s for both legs (H/Q180). The H/Q strength ratio was obtained by dividing the maximum moment of strength of the hamstrings by the maximum moment of strength of the quadriceps. The isokinetic testing protocol was conducted on both legs equally, and the procedures are displayed in Figure 1.

### 2.3. Statistical Analysis

The normality of the distribution was checked using the Kolmogorov–Smirnov test. Arithmetic means and standard deviations were calculated for all included numerical variables.

To determine differences in the studied variables between individuals with and without knee hyperextension, the following statistical procedures were conducted. First, an independent sample *t*-test was conducted on the anthropometric and muscle strength data. Dependent variables included anthropometric indices, the degree of knee hypermobility, and isokinetic parameters of leg musculature, while the grouping variable was the group of hypermobile or nonhypermobile athletes. Additionally, effect sizes (ESs) and confidence intervals were calculated for the same variables for accurately communicating the magnitude of the observed differences, as effect sizes help to understand the practical significance of statistical results beyond mere statistical significance. Cohen’s d effect sizes were interpreted as: below 0.02 = trivial; 0.2–0.6 = small; more than 0.6–1.2 = moderate; and more than 1.2–2.0 = large ES [26].

Second, discriminant analysis with Chi-Square tests with successive roots removed was performed to further assess differences between groups in a multivariate manner or, more precisely, to identify variables that predicted belonging to the group of hypermobile or nonhypermobile athletes. Variables included in the discriminant analysis were anthropometric indices including leg length, shin length, body mass, and body height and strength parameters including H/Q60 and H/Q180, and the grouping variable was hypermobile or nonhypermobile athletes.

To determine the associations between hypermobility indices and other study variables, Pearson’s correlation coefficients were calculated. Moreover, linear multiple regression analysis was performed to determine the multivariate associations between predictors (all anthropometric and strength variables, please see Variables Section) and criteria (hypermobility degree—Hdeg). Prior to the multiple regression analysis, predictors were checked for multicollinearity, and due to a high variance inflation factor (value greater than 10), BMI was not included in the analysis.

The statistical package Statistica 13.5 (Tibco, Inc., Palo Alto, CA, USA) was used for all analyses, and a *p* value lower than 0.05 indicated statistical significance.

## 3. Results

The descriptive statistics and differences between athletes with and without knee hyperextension are shown in Table 1. Significant differences were found in leg and shin length; athletes with hyperextended knees had shorter legs and shins than the athletes without knee hyperextension did. Moreover, regarding the muscle strength parameters, athletes with hyperextended knees had a lower H/Q ratio at an angular velocity of 60°/s.

**Figure 2:**
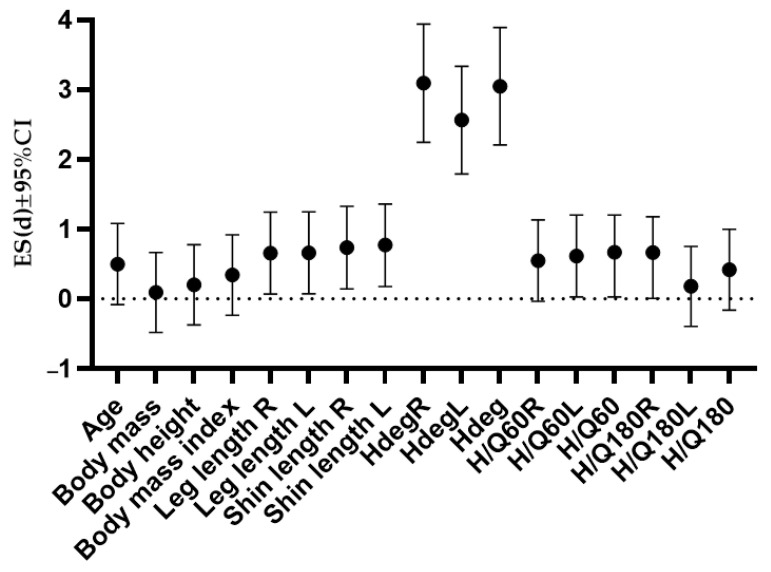
Differences in study variables between athletes with and without knee hyperextension, expressed through Cohen’s d effect sizes. Note: ES—effect size, CI—confidence interval, Hdeg—Degree of knee hypermobility, R—right leg, L—left leg, H/Q60—hamstrings-to-quadriceps ratio at an angular velocity of 60°/s, H/Q180—hamstrings-to-quadriceps ratio at an angular velocity of 180°/s.

When further observing differences between groups, there were differences in the leg (moderate effect size) and shin length (moderate effect size) and H/Q ratio at an angular velocity of 60°/s (moderate effect size). The effect sizes and confidence intervals are presented in Figure 2.

Discriminant analysis and factor structure confirmed the previously mentioned results, as leg and shin length and the H/Q ratio at an angular velocity of 60°/s accounted for classifying athletes into their groups (i.e., hypermobile vs. nonhypermobile). The results of the discriminant analysis and factor structure matrix are presented in Table 2.

The results of the univariate and multivariate associations are presented in Table 3.

A variable H/Q ratio at an angular velocity of 60°/s was associated with the degree of knee hypermobility. Specifically, the results showed that athletes with a lower H/Q ratio at an angular velocity of 60°/s had increased degrees of hypermobility.

## 4. Discussion

Regarding the study’s aims and hypotheses, there are several important findings. First, athletes with hyperextended knees had shorter legs and shins than athletes without knee hyperextension. Second, athletes with hyperextended knees had lower H/Q ratios at an angular velocity of 60°/s than athletes without knee hyperextension did, which was supported by the correlation analysis, as athletes with lower H/Q ratios at an angular velocity of 60°/s had increased hypermobility.

### 4.1. Anthropometric Indices and Hypermobility of the Knee

One of the premises of this research was to determine whether anthropometric characteristics influence knee hypermobility. The expected outcome was that a longer leg and shin due to greater leverage on the knee itself would result in a greater degree of knee hypermobility. However, the results indicated that a greater degree of knee hypermobility is related to a shorter leg and shorter length.

Moreover, by analyzing the differences between the two groups, it was determined that they differed significantly in terms of the anthropometric variables leg length and shin length; for example, the group of hypermobile subjects had an average of 2.5 cm shorter legs than the nonhypermobile subjects did, and there was also a difference in the length of the shin (approx. 2 cm). These data contradict physical postulates, in which the assumption is that a longer leg will have greater leverage, i.e., greater hypermobility, or, in this case, the group of hypermobile subjects will have higher values than other groups [27]. A possible explanation for this result is that a shorter leg can mean a larger cross section of the quadriceps muscle; thus, overextending the leg can increase the strength of the knee [28].

These results can, to some extent, provide a good basis for a more detailed analysis of the anthropometric characteristics of people with hypermobility in a larger sample. It would be particularly interesting to investigate whether lower leg length values also occur in a larger sample of hypermobile subjects, i.e., whether their shorter leg has greater knee hypermobility and whether the size of the cross-section of the quadriceps muscle influences the degree of hypermobility of the same knee.

### 4.2. Isokinetic Indicators and Hypermobility of the Knee

On the basis of the goal of this study, we aimed to demonstrate that people with hypermobile knees differ from people without knee hypermobility in terms of isokinetic indices. Indeed, by analyzing the differences between athletes with and without knee hyperextension in isokinetic indicators, a statistically significant difference is visible in the first isokinetic variable, the one that measures standard isokinetic indicators (60°/s) in such a way that the group of hypermobile subjects had lower values of the H/Q ratio, i.e., lower values of hamstrings strength compared to the group of nonhypermobile subjects.

The findings from our research are to some extent supported by a study in which the H/Q ratio was evaluated in patients with joint hypermobility using an isokinetic device [29]. Specifically, knee extensor muscle strength was substantially lower in the group with joint hypermobility than in the healthy individuals. The study hypothesized that muscular weakness was linked to quadriceps muscle lengthening, pain-related inactivity, joint instability, and proprioception defects. Hypermobility involves collagen tissue with a decreased width and disordered fiber ratio, resulting in a decreased tonus of elastic tissue and a greater risk of traumatic lesions [30]. Loss of soft-tissue strength leads to unstable joints, loss of proprioception, increased risk of traumatic injuries, and decreased activity due to discomfort [29]. Moreover, research on children and adults with joint hypermobility noted changes in muscle activation and coactivation patterns in hypermobile individuals [13]. Specifically, hypermobile individuals had a greater coactivation ratio during isometric knee flexion than the healthy controls did, indicating decreased agonist activation. This might be due to the need to stabilize the hypermobile knee in both the anterior and posterior directions [13].

However, the second isokinetic variable, the one indicating the functional H/Q ratio (180°/s), did not distinguish between the two groups of subjects. The authors of the study noted the twitch (i.e., jerk) of the hamstring muscle at the extreme angle of knee flexion (last 20° of knee flexion), which potentially led to false higher strength values. Namely, in the analysis of isokinetic indices, especially of the functional type, additional focus should be placed on the time and angle at which the maximum values are achieved, especially for the hamstrings, because it was recorded in this research that the maximum value of the hamstrings at 180°/s was achieved in the last parts of the flexion movement, in which the function of the hamstrings, if we look at its stabilizing function on the knee, is completely irrelevant. Such artifacts, if removed, would result in a distortion of the H/Q ratio at higher angular velocities of movement and could affect the overall association between hypermobility and isokinetic indicators.

It is noteworthy that knee hyperextension is a common issue in stroke patients, and it has been linked to femoral cartilage degeneration [31]. Additionally, hyperextension in the stroke population can lead to increased spastic hypertonia [32]. To address this issue, closed kinetic chain exercises are proposed as a potential solution. These exercises have been shown to be effective in reducing spasticity and improving functional outcomes in stroke patients, as well as improving balance and lower extremity muscle activation [32]. This is important to note as further studying knee hyperextension can be beneficial to implement in different settings and conditions.

Overall, the results of this research showed that there was a weak, statistically significant negative correlation between the degree of knee mobility and the H/Q ratio, which indicated that the greater the degree of knee hypermobility was, the lower the strength of the hamstrings. Therefore, it can be theorized that implementing targeted training programs for precisely strengthening hamstring muscles should prevent or ameliorate knee hypermobility disorders. However, these correlations are not large enough to be practically applicable, which means that this issue should be further investigated.

### 4.3. Limitations and Strengths

The main limitation of this research is that it included only male participants, which limits the generalizability of the results on female individuals. Also, the included participants were relatively young, which also limits the applicability of the results in other age groups (i.e., older adults, elderly). This study was performed on a convenient sample, which is one of its limitations. Parameters such as hours of training, type of training, and years of training were not included in the study, which should be addressed in future similar studies. On the other hand, this is among the first studies to measure isokinetic indices of the upper leg muscles in healthy male athletes with knee hypermobility greater than 10° from full extension. This is important to emphasize, as the majority of previous research was conducted on injured athletes and females, while we aimed to include noninjured participants to avoid injury-related muscle imbalances.

## 5. Conclusions

The results of this research showed that athletes with knee hypermobility have weaker hamstring strength and thus a lower H/Q strength ratio at lower angular velocities. Moreover, the H/Q ratio at an angular velocity of 60°/s is associated with the degree of knee hypermobility. These findings suggest that targeted strength training programs for leg muscles should help patients with knee hypermobility. Hence, this research should serve as a guideline for future investigations that include larger sample sizes and athletes of other age groups to be able to draw accurate conclusions about the observed issue.

## Figures and Tables

**Table 1:** Descriptive statistics and differences between athletes with and without knee hyperextension.

Variable	Hyperextended Knee (n = 22)		Nonhyperextended Knee (n = 25)		*t*-Test	
	Mean	SD	Mean	SD	t Value	*p*-Level
Age (years)	22.82	3.22	24.59	3.81	−1.67	0.10
Body mass (kg)	80.40	9.53	79.40	11.88	0.32	0.75
Body height (cm)	180.86	7.24	182.28	6.90	−0.69	0.50
Body mass index	24.52	1.88	23.79	2.33	1.18	0.25
Leg length Right (cm)	87.11	4.11	90.26	5.35	−2.23	0.03
Leg length Left (cm)	87.26	4.20	90.39	5.18	−2.25	0.03
Shin length Right (cm)	40.10	2.89	42.94	4.54	−2.52	0.02
Shin length Left (cm)	39.98	2.92	43.01	4.64	−2.64	0.01
HdegR (°)	12.51	2.21	5.84	2.11	10.57	0.001
HdegL (°)	12.79	1.98	6.80	2.61	8.76	0.001
Hdeg (°)	12.65	1.95	6.32	2.18	10.44	0.001
H/Q60R	0.55	0.08	0.60	0.10	−1.79	0.08
H/Q60L	0.53	0.07	0.58	0.09	−1.97	0.05
H/Q60	0.54	0.07	0.59	0.09	−2.11	0.04
H/Q180R	0.61	0.09	0.67	0.11	−1.88	0.07
H/Q180L	0.64	0.10	0.66	0.12	−0.62	0.54
H/Q180	0.63	0.09	0.67	0.10	−1.34	0.19

Note: Hdeg—Degree of knee hypermobility, R—right leg, L—left leg, H/Q60—hamstrings-to-quadriceps ratio at an angular velocity of 60°/s, H/Q180—hamstrings-to-quadriceps ratio at an angular velocity of 180°/s, SD—standard deviation.

**Table 2:** Discriminant analysis and factor structure matrix according to hypermobility groups.

Chi-Square Tests with Successive Roots Removed				
Eigenvalue	Canonical R	Wilks’ Lambda	Chi-Square	*p* Value
0.65	0.63	0.60	21.13	0.01
Factor Structure Matrix				
Leg length	−0.41			
Shin length	−0.47			
Body mass	0.06			
Body height	−0.13			
H/Q60	−0.39			
H/Q180	−0.25			

Note: H/Q60—hamstrings-to-quadriceps ratio at an angular velocity of 60°/s, H/Q180—hamstrings-to-quadriceps ratio at an angular velocity of 180°/s.

**Table 3:** Univariate and multivariate associations between study variables and degree of knee hypermobility.

Variable	Pearson’s Correlation		Linear Multiple Regression		
	Pearson’s R	*p* Value	β	b	*p* Value
Leg length	−0.24	0.11	−0.56	−0.42	0.05
Shin length	−0.21	0.16	−0.27	−0.25	0.23
Body mass	0.10	0.52	0.23	0.08	0.27
Body height	−0.02	0.83	0.40	0.22	0.21
H/Q60	−0.29	0.04	−0.59	−27.27	0.02
H/Q180	−0.12	0.4	0.41	15.93	0.09

Note: H/Q60—hamstrings-to-quadriceps ratio at an angular velocity of 60°/s, H/Q180—hamstrings-to-quadriceps ratio at an angular velocity of 180°/s, β—standardized regression coefficient, b—non-standardized regression coefficient.

## Data Availability

Data will be available upon reasonable request.
